# Serum ferritin and delirium risk: an integrative genomic analysis of causal inference and multi-tissue regulatory signals

**DOI:** 10.1186/s40246-026-00972-5

**Published:** 2026-04-25

**Authors:** Amirhossein Saed, Mahdi Akbarzadeh, Fatemeh Gohari, Nafiseh Asadrouh, Amir Mohammad Jahromizadeh, Hani Sabaie, Maryam Zarkesh, Mehdi Hedayati, Fereidoun Azizi, Maryam Sadat Daneshpour

**Affiliations:** 1 School of Medicine, Shahid Beheshti University of Medical Sciences, Tehran, Iran; 2 Cellular and Molecular Endocrine Research Center, Research Institute for Endocrine Molecular Biology, Research Institute for Endocrine Sciences, Shahid Beheshti University of Medical Sciences, PO Box: 1985717413, Tehran, Iran; 3 Endocrine Research Center, Research Institute for Endocrine Sciences, Shahid Beheshti University of Medical Sciences, Tehran, Iran

**Keywords:** Delirium, Ferritin, Mendelian randomization, Colocalization, Multi-omics

## Abstract

**Background:**

Delirium is an acute neuropsychiatric syndrome characterized by disrupted attention and cognition, often triggered by systemic inflammation and physiological stress. Elevated serum ferritin is frequently observed in patients with delirium. Since ferritin couples iron handling to inflammatory signaling during acute-phase responses, it remains unclear whether genetically influenced baseline serum ferritin is a modifiable causal risk factor for delirium, or whether ferritin elevations observed during illness mainly reflect downstream systemic states leading to brain network failure. We used genetic triangulation to assess baseline causality and identify regulatory mechanisms influencing delirium susceptibility.

**Results:**

Using harmonized GWAS summary statistics for ferritin (GCST90270865; *N* = 270,794) and delirium (GCST90473243; 8461 cases, 449,979 controls), we found no evidence that genetically proxied increases in ferritin causally raise delirium risk (primary MR: IVW random-effects OR = 1.09 per 1/SD ferritin, 95% CI 0.93–1.26; *p* = 0.282). Genome-wide overlap was limited, with weak, non-significant cross-trait genetic correlation and minimal shared polygenic signal. Mechanistic follow-up at the locus level across multi-tissue QTL resources identified widespread ferritin-linked cis-QTL signals (255 probes, 126 genes), while delirium showed sparse mediator signals (four probes, three genes), all on chromosome 19. At 19q13, ferritin strongly colocalized with an *APOE* plasma pQTL (PP.H4 = 0.999; SuSiE PP.H4 ≈ 1.00), whereas delirium colocalized with a cortex *CEACAM19* eQTL (PP.H4 = 0.9983). Outside 19q13, ferritin colocalized with iron regulation signals at *SLC11A2* whole-blood eQTL (PP.H4 = 0.853) and *TF* liver sQTL (PP.H4 = 0.966), with no evidence of ferritin–delirium colocalization.

**Conclusions:**

Genetic evidence does not support baseline ferritin as a primary, modifiable causal factor for delirium risk. Instead, inherited susceptibility appears locus-specific and seems to align with brain regulatory mechanisms, including a cortical signal at 19q13, distinct from iron homeostasis. These findings emphasize the need for mechanistic and preventive research targeting brain-relevant pathways other than systemic iron management for delirium prevention.

**Graphical Abstract:**

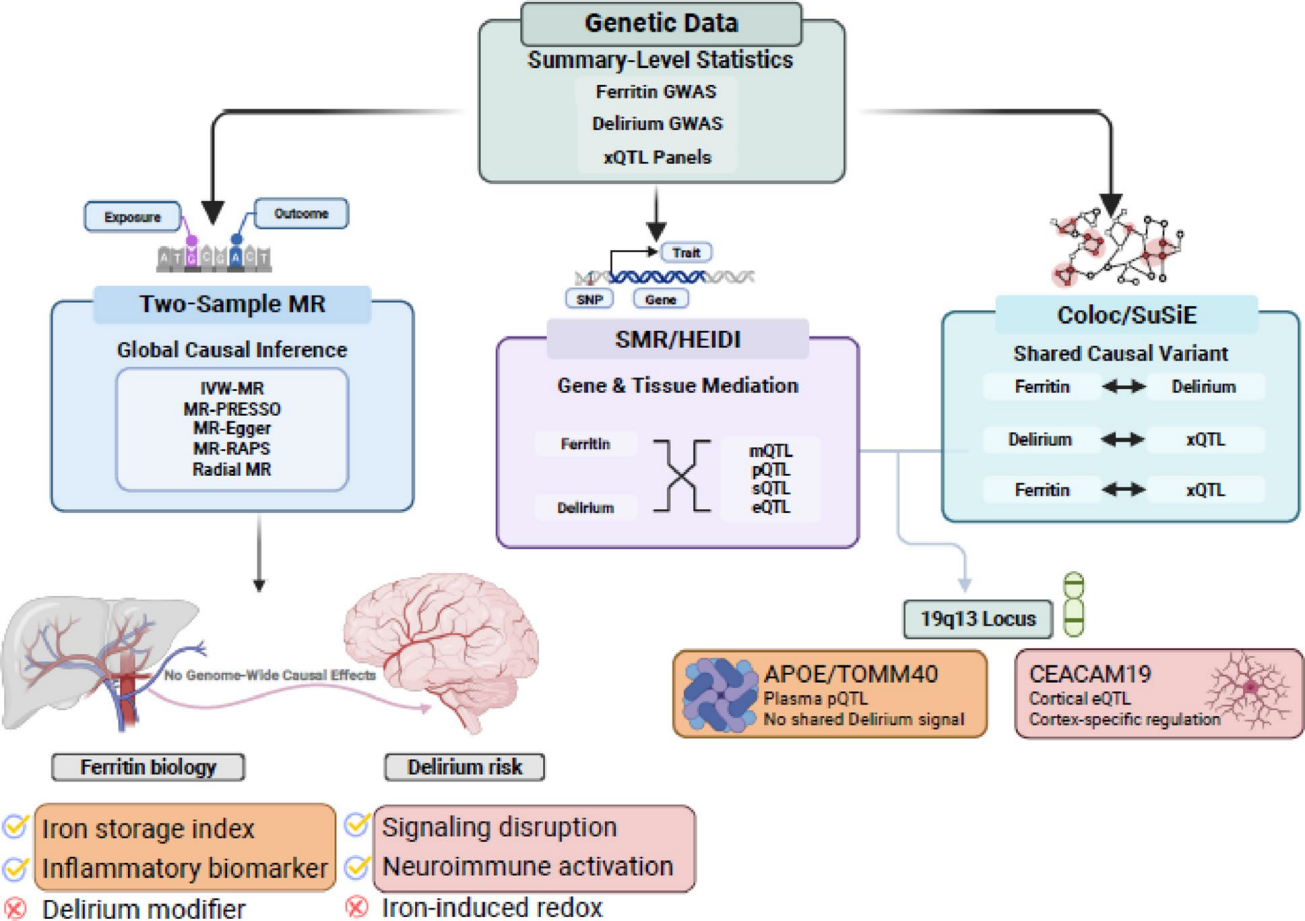

**Supplementary Information:**

The online version contains supplementary material available at10.1186/s40246-026-00972-5.

## Background

Delirium is a severe neuropsychiatric syndrome characterized by an acute state of disruption in attention and cognition, and is among the most frequent complications during hospitalization in older adults, often linked to longer stays, functional and cognitive decline, and an increase in mortality [1, 2]. Delirium’s heterogeneous presentation is shaped by interactions between the patient’s baseline vulnerabilities (predisposing factors), including age, comorbidities, and genetic risk factors, which influence the brain’s resilience to acute precipitating factors like surgery, anaesthesia, perioperative medications, infection, and other systemic insults. Currently, delirium’s pathophysiology is viewed as a disorder of large-scale brain network function, where systemic inflammation, metabolic stress, and altered neurotransmission affect glial and vascular interfaces, causing temporary but significant network disintegration [2, 3]. Both mechanistic and neurophysiological evidence point to disruptions in functional connectivity as a hallmark of delirium, while inflammatory models highlight the blood–brain barrier (BBB) responsivity and microglial activation as key pathways through which peripheral inflammation leads to acute cognitive impairment [4, 5].

Systemic iron homeostasis is maintained by the hepcidin-ferroportin axis, which controls iron absorption, recycling, and mobilization from macrophages and hepatocytes, while excess iron is stored safely in ferritin complexes, sequestered away from biological processes [6, 7]. Serum ferritin is used clinically as an indicator of iron status, and it also acts as an acute-phase reactant that can rise substantially in response to infection, trauma, malignancy, and cytokine-driven inflammation, making it less reliable as a measure of true iron stores in acutely ill patients [8, 9]. Its role is especially relevant in delirium, given that delirium is a syndrome of acute brain dysfunction often triggered by systemic inflammation, where iron-dependent oxidative injury remains biologically plausible: iron participates in redox reactions, promoting lipid peroxidation and ferroptosis, an iron-dependent form of regulated cell death increasingly discussed in acute and perioperative neurocognitive vulnerability, though more supported by experimental evidence than in human delirium [4, 10]. A key uncertainty is whether the frequent co-occurrence of delirium and high serum ferritin reflects a modifiable upstream pathway in systemic iron biology or merely an acute-phase biomarker of illness severity that is associated with, but not causally responsible for, brain network failure.

Observational studies frequently report associations between ferritin levels and delirium in perioperative and acute care settings. However, understanding the direction and nature of these relationships can be complex, with some evidence suggesting non-linear patterns that may indicate competing mechanisms rather than a straightforward causal effect. In this context, genetic triangulation methods can help clarify the mechanisms underlying the ferritin-delirium link, offering insights that are hard to achieve with observational data alone [11].

Objectives: In the current study, we aimed to determine whether baseline ferritin set-points constitute a plausible causal and potentially actionable pathway for delirium using Mendelian randomization (MR), and to localize tissue- and locus-specific regulatory mechanisms using complementary genetic tools.

## Methods

### Study design

We used genome-wide, gene, and locus-level inference to explore the genetic links between ferritin biology and delirium susceptibility. Methods included linkage disequilibrium score regression (LDSC) for polygenicity analyses [12], two-sample MR for exposure-outcome relationships, summary-data-based Mendelian randomization (SMR) with heterogeneity in dependent instruments (HEIDI) testing for cis-regulated mediators, Bayesian colocalization to assess shared causal variants, and Sum of Single Effects (SuSiE) fine-mapping for multi-signal regions.

### Data sources and eligibility

We used de-identified aggregate statistics from public sources; no new individual data were collected. Ferritin exposure data were obtained from the GWAS Catalog (accession GCST90270865) [13], provided on GRCh38 with forward-strand alleles and rsIDs mapped to dbSNP 151, with effect estimates on the original scale. Delirium data were from GWAS Catalog (GCST90473243) [14], harmonized on GRCh38 using dbSNP rsIDs. The GWAS and QTL resources involved mixed-sex cohorts mainly of European ancestry. No sex-stratified data were available for ferritin or delirium, so analyses estimate average effects across sexes.

Molecular QTL resources for SMR/HEIDI were accessed via the SMR Portal (yanglab.westlake.edu.cn) on 25 September 2025, including GTEx v8 eQTL/sQTL (liver, brain, pituitary, spleen, kidney) [15], eQTLGen Whole Blood [16], MetaBrain brain eQTL [17], GoDMC blood mQTL [18], and Fenland plasma pQTL [19] and INTERVAL plasma pQTL [20]. These tissues were not used to redefine the primary exposure; rather, serum ferritin remained the exposure phenotype throughout causal inference analyses, while multi-tissue QTL datasets were used for mechanistic follow-up to localize tissue- and locus-specific regulatory signals relevant to ferritin biology and delirium susceptibility. Liver, kidney, and blood were included because they are central to ferritin biology and systemic iron regulation, spleen to identify possible immunological mediation, and brain cortex as well as pituitary because delirium is a syndrome of acute brain dysfunction, making brain-related tissues necessary for mechanistic analyses. For colocalization, when SMR Portal panels were not publicly downloadable, we used other publicly available matched tissues to enable window-based extraction. These publicly available resources included GTEx v8 per-tissue eQTL summary statistics, GTEx v10 significant sQTL panels, eQTLGen Whole Blood (GRCh37), INTERVAL plasma pQTL releases available via OpenGWAS, and GoDMC Blood mQTL (GRCh37).

The eligibility criteria for inclusion as a data source required clearly defined effect and non-effect alleles, per-variant effect sizes with standard errors or p-values, rsID availability, a documented genome build, and sufficient genome-wide variant coverage for regional extraction.

The linkage disequilibrium (LD) reference was 1000 Genomes Phase 3 EUR (1KG-EUR). HEIDI used the portal’s internal 1KG-EUR LD, and other analyses used the external 1KG-EUR LD.

All datasets were used in accordance with their respective public or consortium data-use terms, and the study was approved by the local ethics committee at the Research Institute for Endocrine Sciences, Shahid Beheshti University of Medical Sciences (Research Approval Code: 43017675).

### Genetic data processing

Variant-level preprocessing was applied identically to exposure and outcome summary statistics prior to analysis. We retained biallelic A/T/C/G SNPs with rsID, required EAF (or MAF) ≥ 0.005, excluded indels and multi-allelic loci, and applied INFO ≥ 0.8 when available.

We standardized fields (A1, A2, effect, SE, p, EAF) and removed rows with non-numeric or out-of-range values. Duplicate rsIDs were collapsed by retaining the row with the smallest p-value. We preserved forward-strand orientation, retained palindromic variants only when the frequency resolved strand ambiguity, and excluded records with missing essential fields. Post-QC exports included an MR-ready table and the SMR eight-column GCTA format.

**Fig. 1:**
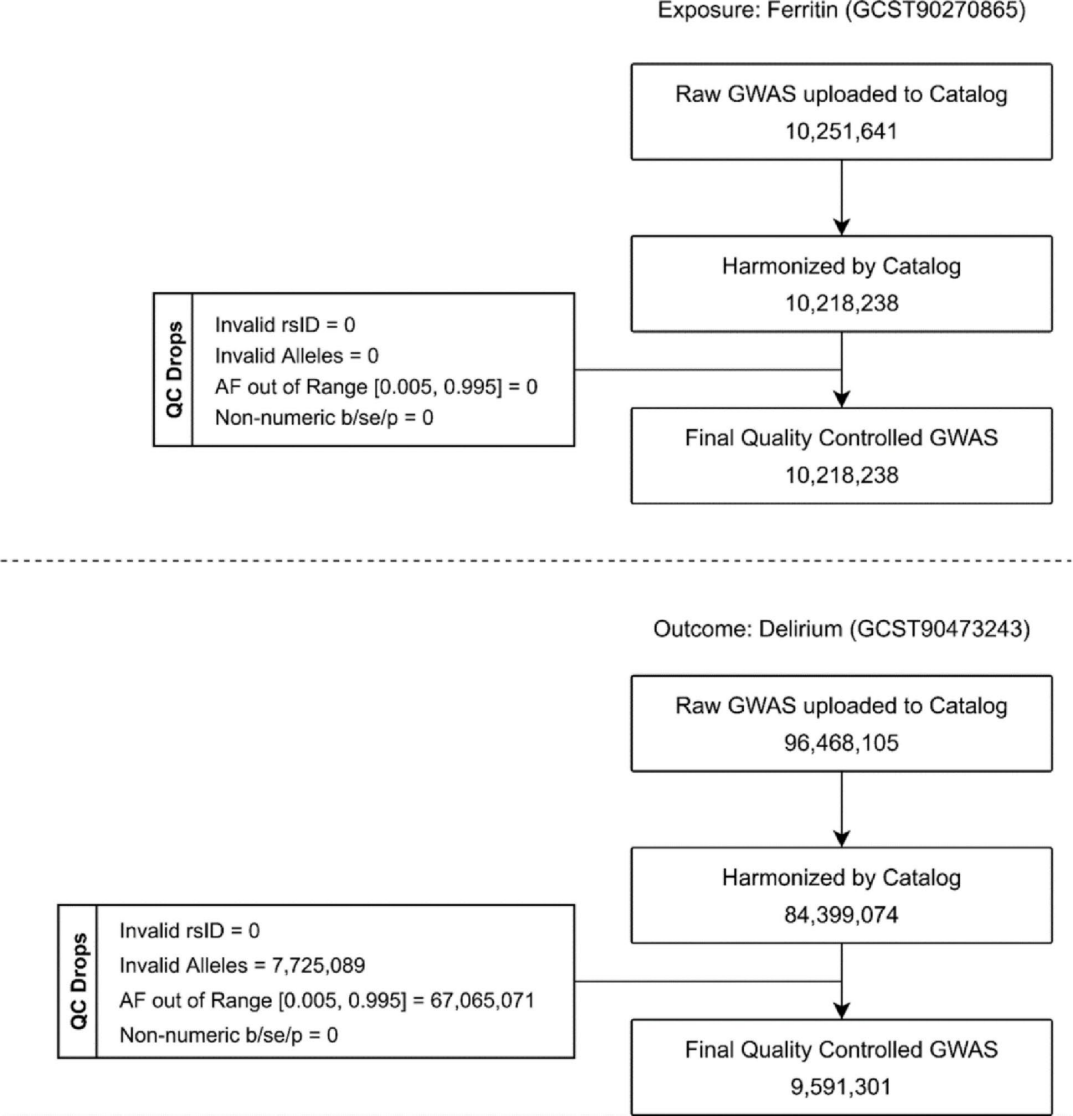
Data overview and quality control (QC) flow. *QC flowcharts for ferritin (top) and delirium (bottom) display the number of sites submitted to the GWAS Catalog*,* those successfully harmonized*,* inputs to variant-level QC*,* and final unique SNPs retained*,* with side calls for drop reasons (invalid rsID*,* alleles*,* allele frequency*,* INFO < 0.8*,* non-numeric)*

Public GWAS and QTL sources were used as provided, i.e., where QTL panels were GRCh37 and GWAS were GRCh38, we relied on rsID identity for variant pairing and did not perform panel-wide liftOver. All downstream harmonizations enforced consistent effect-allele orientation and removed mismatches. QC audits recorded the retained counts and drop reasons, as presented in Fig. 1.

### LD score regression (LDSC)

We used LDSC to estimate SNP heritability and genetic correlation, munging summary statistics for HapMap3 SNPs (MAF ≥ 0.01) and applying default allele checks. The major histocompatibility complex region (chr6:26–34 Mb) was excluded from all LDSC regressions.

Univariate LDSC was used to estimate observed-scale SNP heritability and the LDSC intercept for each trait. Delirium h² was converted to the liability scale using K_pop = 0.20 and K_samp = 8461/458,440 ≈ 0.0185. Bivariate LDSC estimated rg and the cross-trait intercept. We used the precomputed 1000 Genomes Phase 3 EUR LD scores eur_w_ld_chr (HapMap3 SNPs; HLA removed on chr6) and the HapMap3 regression weights excluding the MHC weights.hm3_noMHC (plus w_hm3.snplist) from the LDSC data repository (accessed 06 Oct 2025), with block jackknife SEs and default LDSC outlier handling.

### Two-sample MR

We performed two-sample MR with ferritin as the exposure and delirium as the outcome, using harmonized summary statistics. Instruments were genome-wide significant variants (*p* < 5 × 10⁻⁸) clumped at r² < 0.001 within a 10 Mb window using a EUR LD reference, retaining the lead SNP per LD region.

The primary estimator was inverse-variance-weighted (IVW) under a random-effects model. Instrument strength (per-SNP and mean F), heterogeneity (Cochran’s Q), and directional pleiotropy (MR-Egger intercept) were assessed.

Outlier-robust inference utilized MR-PRESSO (global/outlier/distortion) by comparing pre- and post-correction estimates, employing robust estimators (weighted median/mode). Directionality was evaluated using Steiger filtering, the influence of individual instruments via leave-one-out analyses, and small-study asymmetry patterns via funnel plots.

We performed Radial MR (modified second-order weights and exact random-effects) to evaluate influential instruments and residual heterogeneity. MR-RAPS was also attempted (with/without over-dispersion; robust and non-robust) to assess small-effect idiosyncrasies. Radial/MR-RAPS results are reported only as sensitivity checks.

Delirium effects were modelled on the log-odds scale and reported as odds ratios with 95% confidence intervals. The exposure scale was per 1/SD increase in inverse-rank-normalized ferritin (sex-specific IRNT in the discovery GWAS) and was used consistently.

The primary inference relied on IVW random-effects, with sensitivity analyses used to contextualize heterogeneity and pleiotropy, and two-sided p-values employed throughout. If Cochran’s Q was found significant but the MR-Egger intercept was not, this was treated as possible non-directional pleiotropy/heterogeneity. Robust estimators and Radial MR then arbitrated the stability of the main conclusions.

### SMR/HEIDI across tissues

We conducted SMR with the HEIDI heterogeneity test using the SMR Portal to evaluate whether cis-regulated molecular traits are associated with GWAS signals for serum ferritin and delirium [21]. GWAS summary statistics were provided in the portal-required format (SNP, A1, A2, freq, b, se, p, n), with QTL panels as listed above.

For each probe or gene, the portal used the top cis-QTL SNP as the instrument and tested the association between the molecular trait and the GWAS trait using summary statistics. Analyses used cis windows of ± 2 Mb, with eligibility based on the portal’s top cis-QTL significance filter.

We used the portal defaults, including the SMR significance settings for instrument selection (peqtl-smr = 5 × 10⁻⁸), HEIDI configuration (nsnp range 3–20), LD pruning (r² ≤ 0.9), and allele-frequency mismatch filters, while setting the GWAS MAF input to 0.005 to match upstream GWAS preprocessing.

All SMR output files were then post-processed using a unified pipeline with multiple-testing correction applied at the panel level defined by qtl_name (i.e., separately for each QTL panel within each tissue × omics stratum), with the Bonferroni threshold computed as p_Bonf = 0.05/M, where M denotes the number of unique probes tested in that qtl_name panel. Probes were retained as SMR/HEIDI-consistent if they satisfied p_SMR ≤ p_Bonf, showed no evidence of heterogeneity (p_HEIDI ≥ 0.01), and were HEIDI-testable (nsnp_HEIDI ≥ 3). Probes failing frequency checks or lacking ≥ 3 candidate SNPs for HEIDI were excluded as per the portal output. For reporting, probe-level results were aggregated to gene-level summaries and to trait × tissue × omics tables.

### Bayesian colocalization and SuSiE-based fine-mapping

The primary analysis used single-signal coloc.abf to estimate posterior probabilities for hypotheses H0-H4, with PP.H4 indicating a shared causal variant and PP.H3 indicating distinct causal variants in linkage disequilibrium. Windows were centered on the target gene or locus (default ± 250 kb) and expanded to ± 500 kb for the *APOE*/19q13 region to account for dense LD and known multi-signal architecture. Inputs included effect estimates and standard errors per variant (or signed effects with p-values where applicable), alleles, and MAF when available. Variants were harmonized to a consistent effect-allele orientation, and discordant or ambiguous alleles (including palindromic variants without reliable frequency data) were excluded. For each locus pair, LD matrices for intersecting SNPs were generated from 1KG-EUR. Colocalization priors were set to p1 = p2 = 1 × 10⁻⁴ and p12 = 1 × 10⁻⁵, with sensitivity to p12 evaluated using a grid where applicable. PP.H4 ≥ 0.80 was interpreted as strong evidence for sharing, 0.50–0.80 as moderate evidence for sharing, and a high PP.H3 as evidence of distinct causal signals.

Since coloc.abf assumes at most one causal variant per trait within a window, loci with evidence of multiple signals or PP.H3-dominant outcomes in multi-signal areas were further analyzed using SuSiE fine-mapping. Each trait was fine-mapped with susie_rss (L = 5, refine = TRUE) followed by component-wise colocalization via coloc.susie. We identified the component pair(s) with the strongest support and the corresponding posterior evidence for sharing. When SNP-level posteriors were unavailable for a top component pair, we used a posterior-overlap proxy based on component SNP posterior vectors to summarize overlap. Locus pairs not meeting minimal criteria (e.g., insufficient overlapping SNPs after QC) were excluded from primary analysis and noted as not meaningfully testable with the current panels or windows.

### Software, versions, and reproducibility

Analyses were conducted on Windows 11 (Microsoft Corporation, USA, build 26200.6725) with Microsoft standard WSL2 (Microsoft Corporation, USA, version 6.6.87.2), Ubuntu 24.04.3 LTS (Canonical Ltd., UK), R version 4.5.1 (R Foundation for Statistical Computing, Austria), and Python version 3.11.9 (Python Software Foundation, USA). Colocalization and fine-mapping were performed using the coloc package version 5.2.3, including coloc.abf and coloc.susie (R Foundation for Statistical Computing, Austria) with per-locus LD matrices from 1000 Genomes Phase 3 EUR. VCF inspection and indexing were performed using bcftools version 1.10.2 and htslib/bgzip/tabix 1.10.2 (Genome Research Ltd., UK). Two-sample MR used the R packages TwoSampleMR version 0.6.21 and ieugwasr version 1.1.0 (R Foundation for Statistical Computing, Austria). Pleiotropy and outlier analyses were performed using MR-PRESSO version 1.0 in R. SMR/HEIDI analyses were run via the SMR Portal and SMR software by Yang Lab, Westlake University, China (accessed 25 September 2025). LDSC analyses used ldsc version 2.0.0 (Bulik-Sullivan and Finucane groups, Broad Institute, USA) with 1KG-EUR LD scores reported above. Data wrangling and visualization were performed using the R packages data.table version 1.17.8, dplyr version 1.1.4, and ggplot2 version 4.0.

ChatGPT and Grammarly AI were used only for language editing purposes and all scientific decisions and final wording remain the authors’ responsibility.

## Results

### Data overview and QC

We analysed GWAS Catalog-harmonized summary statistics for ferritin (GCST90270865) and delirium (GCST90473243), both aligned to GRCh38 with rsIDs mapped to dbSNP b151. Ferritin comprised 270,794 European-ancestry participants (array-based GWAS, imputed), and delirium comprised 8,461 non-Finnish European cases and 449,979 controls from the UK Biobank whole-genome sequencing (ICD-10 F05). GWAS Catalog harmonization metrics showed a high harmonization success rate for ferritin (99.67% of carried-forward sites) and lower for delirium (87.49%), consistent with WGS density. Post-harmonization variant-level QC is presented in Fig. 1.

### Heritability and genetic correlation (LDSC)

After hapMap3 processing and LD-score merging, delirium retained 1,130,371 SNPs, with an observed-scale heritability (h²) of 0.0026 (SE 0.0011), intercept of 1.0183 (SE 0.0074), mean χ² of 1.0422, λGC of 1.0375, and an attenuation ratio of 0.4339 (SE 0.1751), indicating limited inflation and minimal sample overlap. Assuming a baseline prevalence (K) of 0.20 and a GWAS case fraction (P) of 0.0185, heritability on the liability scale was 0.0469.

For ferritin, LDSC estimated an observed-scale h² of 0.0635 (SE 0.0058), intercept of 1.0761 (SE 0.0113), mean χ² of 1.4315, λGC of 1.2831, and attenuation ratio of 0.1763 (SE 0.0262), using 1,159,589 SNPs.

Delirium’s low heritability suggests limited common-variant signal, mild inflation, and minimal overlap. Ferritin’s higher heritability indicates a moderate common-variant contribution, with inflation mainly due to polygenicity.

The genetic correlation between ferritin and delirium, estimated from 1,159,589 SNPs via cross-trait regression, was rg = 0.122 (SE = 0.1025; *p* = 0.2338), indicating a weak, non-significant positive correlation. The cross-trait LDSC intercept was 0.0024 (SE 0.0051), suggesting minimal overlap between datasets.

### Primary causal inference

For two-sample MR, after clumping at r²<0.001 within 10 Mb, 70 independent ferritin instruments were retained (mean F = 78.76). The IVW random-effects estimate showed no association of genetically proxied ferritin with delirium (β = 0.082, SE = 0.076; *p* = 0.282), corresponding to OR = 1.09 (95% CI 0.93–1.26) per 1/SD increase in inverse rank-normalized ferritin. This effect size is negligible and indicates no material causal impact.

Heterogeneity was present (Q_IVW = 96.995, df = 69, *p* = 0.015; Q_Egger = 96.361, df = 68, *p* = 0.013), but the MR-Egger intercept did not differ from zero (− 0.00348, SE = 0.005; *p* = 0.506), indicating no directional pleiotropy. Sensitivity analyses were directionally consistent: weighted median β = 0.090 (SE = 0.097; *p* = 0.356) and MR-Egger slope β = 0.162 (SE = 0.141; *p* = 0.257).

MR-PRESSO detected outliers (global *p* = 0.017); the outlier-corrected IVW was nominal (β = 0.120, SE = 0.059; *p* = 0.0445; OR = 1.13, 95% CI 1.01–1.26) with a non-significant distortion test (*p* = 0.705). Steiger directionality supported ferritin→delirium (variance explained ≈ 2.06% vs. ≈0.022%; *p* = 0), noting the large exposure vs. relatively small outcome case count.

A pre-specified sensitivity set (r² < 0.001, 10 Mb; additional *p* < 5 × 10⁻⁸+proximity trimming) yielded ~ 50 instruments to which Radial MR was applied and detected no outliers and no residual heterogeneity (Q = 12.8 on 49 df; *p* = 1.0). The Radial MR-Egger intercept was non-significant (classic scale intercept = − 0.00038; *p* = 0.74). Radial IVW estimates were β ≈ − 0.235 (SE = 0.043–0.046; *P* = 1.5e−6–3.6e−7). MR-RAPS did not converge reliably; therefore, the results were not used for inference. Consistent with the very low residual heterogeneity and limited leverage, these results do not replace the pre-specified 70-IV primary analysis and do not alter the overall null inference. Overall, these results do not support a convincing global causal effect; detailed estimates and diagnostics are provided in Table 1 with full results available as supplementary information (Tables S1, S2, Figures S1–5).

**Table 1:** MR estimates and diagnostics

Category	Estimator	β	SE	*p*	OR	OR 95% CI	Notes
Primary estimate	IVW (random-effects)	0.082	0.077	0.282	1.086	0.93–1.26	nsnp=70
Sensitivity estimate	Weighted median	0.090	0.097	0.356	1.094	0.90–1.32	
Sensitivity estimate	MR-Egger slope	0.162	0.141	0.257	1.176	0.89–1.55	
Sensitivity estimate	Radial IVW	− 0.235	0.043	1.46 × 10⁻⁶	0.79	0.73–0.86	nsnp=50 No outliers; Q=12.8 (49 df), *p*=1.0
Outlier-robust estimate	MR-PRESSO (outlier-corrected IVW)	0.120	0.059	0.045	1.127	1.00–1.26	Global *p*=0.017 Distortion *p*=0.705
Diagnostics	Heterogeneity (IVW)	–	–	0.015	–	–	Q=96.995, df=69
Diagnostics	Heterogeneity (MR-Egger)	–	–	0.013	–	–	Q=96.361, df=68
Diagnostics	MR-Egger intercept	− 0.003	0.005	0.506	–	–	No directional pleiotropy
Diagnostics	Radial Egger Intercept	− 0.489	0.265	0.072	–	–	Non-significant
Diagnostics	Steiger directionality	–	–	0.000	–	–	r2_exposure=0.021 r2_outcome=0.000
Robustness	MR-RAPS	–	–	–	–	–	Did not converge

Primary and sensitivity estimates, plus diagnostics for ferritin→delirium MR. IVW random-effects were primary; robust estimators included the weighted median and MR-Egger slope. Outlier-robust inference uses MR-PRESSO (global/outlier/distortion). Diagnostics include heterogeneity (Cochran’s Q, IVW, and Egger), MR-Egger intercept, and Steiger directionality

### Transcriptomics mediation analysis

Applying Bonferroni-corrected SMR with HEIDI filtering across the full panel inventory (43 tissue-omics QTL panels per trait), Ferritin showed widespread cis-QTL signals, whereas Delirium exhibited a sparse profile. Under the canonical filters (panel-wise Bonferroni within each qtl_name, p_HEIDI ≥ 0.01, and nsnp_HEIDI ≥ 3), Ferritin yielded 255 unique probes mapping to 126 genes (344 probe-panel records), with signals distributed across six tissues and all four omics layers. The largest numbers of passing probes were observed in blood mQTL (40 probes) and brain mQTL (35 probes), alongside strong blood eQTLGen support (23 probes; Supplementary Table S1). The most significant Ferritin associations included *SLC11A2* in blood eQTLGen (best p_SMR = 3.74 × 10⁻¹⁹; p_HEIDI = 0.0169) and *APOE* in blood pQTL (p_SMR = 5.21 × 10⁻⁸; p_HEIDI = 0.589). TF also showed a liver sQTL signal meeting the same filters (p_SMR = 2.93 × 10⁻⁵; p_HEIDI = 0.418).

In contrast, Delirium retained only three genes across four panels (blood pQTL, brain eQTL, brain mQTL, pituitary eQTL). These comprised *APOE* in the blood pQTL (p_SMR = 1.01 × 10⁻¹³; p_HEIDI = 0.055), TOMM40 in the brain mQTL (p_SMR = 1.67 × 10⁻⁸; p_HEIDI = 0.310), and *CEACAM19* in the brain and pituitary eQTL (p_SMR = 3.89 × 10⁻⁶ and 2.02 × 10⁻⁵). Notably, all Delirium-retained signals mapped to chromosome 19. Gene-level aggregation identified *APOE* as the only gene supported in both traits under these strict filters (Table 2). Full SMR and HEIDI results can be found in supplementary Tables S3–10 and supplementary Figures S6–9.

**Table 2:** SMR/HEIDI Bonferroni-significant probe counts and top hits by trait, tissue, and omics layer

Trait	Tissue	Omics	Probes	p_Bonf	m	k	TopGene	TopSNP	Top_pSMR
Ferritin	Blood	eQTL	11,110	4.50E−06	52	23	*SLC11A2*	rs1095998	3.74E−19
Ferritin	Blood	mQTL	13,148	3.80E−06	82	40	TEX14	rs8082334	2.48E−17
Ferritin	Blood	pQTL	2229	2.24E−05	19	10	MPO	rs75394768	6.68E−12
Ferritin	Blood	sQTL	1925	2.60E−05	13	10	MS4A7	rs950803	2.75E−08
Ferritin	Brain	eQTL	32,707	1.53E−06	55	21	RAD51C	rs67014405	9.35E−14
Ferritin	Brain	mQTL	14,598	3.43E−06	106	35	HSF5	rs2240719	4.30E−17
Ferritin	Brain	sQTL	25,406	1.97E−06	49	19	NOD1	rs2190504	8.08E−20
Ferritin	Kidney	eQTL	262	1.91E−04	2	1	BTNL3	rs72494581	8.01E−05
Ferritin	Kidney	sQTL	460	1.09E−04	3	2	NOD1	rs2709801	1.95E−08
Ferritin	Liver	eQTL	1560	3.21E−05	11	6	TSPOAP1	rs8263	1.53E−08
Ferritin	Liver	sQTL	1192	4.20E−05	13	11	TMPRSS6	rs877908	4.09E−10
Ferritin	Pituitary	eQTL	2407	2.08E−05	19	10	ORMDL1	rs6741074	1.25E−15
Ferritin	Pituitary	sQTL	2047	2.44E−05	11	8	NOD1	rs2190504	8.46E−12
Ferritin	Spleen	eQTL	2968	1.69E−05	17	9	SORD	rs4774514	7.40E−13
Ferritin	Spleen	sQTL	2001	2.50E−05	8	8	NOD1	rs2529440	2.15E−18
Delirium	Blood	pQTL	2227	2.25E−05	4	1	*APOE*	rs814573	1.01E−13
Delirium	Brain	mQTL	14,588	3.43E−06	3	1	TOMM40	rs59007384	1.67E−08
Delirium	Pituitary	eQTL	2402	2.08E−05	1	1	*CEACAM19*	rs714948	2.02E−05

For each trait, tissue, and omics level, ‘Probes (M)’ indicates tested probes, with p_Bonf = 0.05/M as the Bonferroni threshold. ‘m’ is the number of SMR-significant probes (p_SMR ≤ p_Bonf), while ‘k’ is the subset passing HEIDI (p_HEIDI ≥ 0.01; nsnp_HEIDI ≥ 3). The TopGene/TopSNP/Top_pSMR columns show the smallest p_SMR among HEIDI-pass probes; ‘NA’ indicates no HEIDI-passing probe exists in that stratum. Rows for delirium tissue × omics combinations without hits are omitted

### Locus-level variant sharing

For ferritin, Bayesian colocalization supported strong sharing with an *APOE* plasma pQTL signal (SomaLogic prot-a-131) within the 19q13 window (chr19:44,421,094 − 45,421,094; GRCh38), with PP.H4 = 0.999 and PP.H3 = 0.00105, and the posterior mass concentrated on two variants (rs1065853 and rs7412). In the confirmatory SuSiE-coloc analysis, component-pair colocalization was consistent with high posterior support for sharing at this locus (component-level PP.H4 = 1.00), with leading hits including rs106433 and rs1065853 (Table 3). Ferritin also colocalized with *SLC11A2* whole-blood eQTL (eQTLGen), showing PP.H4 = 0.853 (with PP.H3 = 0.147) across 3,997 overlapping SNPs, and a 95% credible set of 21 variants; and with a TF liver sQTL (GTEx v10), showing PP.H4 = 0.966 (with PP.H3 = 0.0341) across 995 overlapping SNPs.

For delirium at 19q13, colocalization supported strong sharing with a *CEACAM19* cortex eQTL (GTEx v8), with PP.H4 = 0.9983 and PP.H3 = 0.0010 (5 overlapping SNPs), and posterior mass dominated by 19:44920738:C: T and rs4862277. In contrast, multiple *APOE*-linked molecular signals did not colocalize with delirium: delirium versus the *APOE* plasma pQTL showed PP.H3 ≈ 1.00 with PP.H4 ≈ 2.65 × 10⁻⁴¹, and delirium versus *APOE* brain sQTL signals showed PP.H3 = 1.00 with extremely small PP.H4 values (Cortex: 1.74 × 10⁻²²; BA9: 7.09 × 10⁻⁷¹), indicating distinct causal variants despite locus overlap. Finally, ferritin GWAS versus delirium GWAS within the same 19q13 window likewise supported distinct causal variants (PP.H3 ≈ 1.00, PP.H4 ≈ 3.69 × 10⁻¹¹²), and SuSiE-coloc did not identify any high-probability shared components (maximum component-wise PP_shared ≈ 5.9 × 10⁻⁴).

Beyond 19q13, additional loci evaluated (including *TOMM40*, *NEBL*, and *METTL25*) showed no evidence of colocalization under the available panels and windows; some comparisons couldn’t be meaningfully tested in the intended tissue/omics context due to a lack of suitable QTL signals or overlapping variants. Colocalization and SuSiE results are available in supplementary Tables S11–16 and supplementary Figures S10–12.

**Table 3:** Key coloc.abf and SuSiE fine-mapping at 19q13

Locus	Pair	Coloc PP.H4	Coloc PP.H3	SuSiE summary	Overall_call
19q13 (*APOE* region)	Ferritin GWAS ↔ *APOE* plasma pQTL (SomaLogic prot-a-131)	0.999	0.00105	1 ↔ 1: rs106433 (PP.H4=1.0) 3 ↔ 3: rs1065853 (PP.H4=1.0)	Shared (coloc.H4≈0.999; SuSiE supports)
19q13 (*APOE* region)	Delirium GWAS ↔ *APOE* plasma pQTL	2.65e-41	~1.00	All component pairs PP.H4=0; PP.H3≈1.0 (no sharing)	Distinct (coloc.H3≈1; SuSiE: no sharing)
19q13 (*APOE* region)	Delirium GWAS ↔ *APOE* sQTL (Brain Cortex; proxy panel)	1.74e-22	~1.00	Component-level sharing reported (PP≈0.996–1.000; lead SNPs include rs440446, rs7259620)	coloc.H3-dominant; SuSiE×SuSiE suggests component-level overlap
19q13 (*APOE* region)	Delirium GWAS ↔ *APOE* sQTL (BA9; proxy panel)	7.09e-71	~1.00	Shared component reported (PP≈0.997; lead variant 19:44905910:C: G; discordant direction)	coloc.H3-dominant; SuSiE×SuSiE suggests shared component
19q13 (*CEACAM19* region)	Delirium GWAS ↔ *CEACAM19* cortex eQTL	0.998	0.001	SuSiE not applied/failed (very small overlap SNP set)	Shared (coloc.H4≈0.998; very small SNP set)
19q13 (*APOE* region)	Ferritin GWAS ↔ Delirium GWAS (*APOE* window)	3.69e-112	1	max PP≈5.9 × 10⁻⁴ (no shared components)	Distinct (H3≈1; no shared components)

Summary of coloc.abf and SuSiE fine-mapping at 19q13. PP.H4 shows support for shared causal variants; PP.H3 indicates distinct variants in LD. SuSiE results are included for multi-signal regions

## Discussion

This study implements phenotype-level causal inference using two-sample MR, cis-mediator screening with SMR/HEIDI, and locus-level variant-sharing analyses via Bayesian colocalization with SuSiE fine-mapping to explore a clinically significant problem: whether elevated ferritin indicates a modifiable upstream iron pathway that increases delirium risk, or if it is mainly a stress-induced biomarker of systemic stress leading to acute brain network dysfunction [1, 2]. Across methods, genetically proxied ferritin did not show a convincing causal effect on delirium risk, and LDSC analysis revealed weak, non-significant genetic correlation. Instead of a widespread genome-wide signal, the genetic evidence pointed to specific loci and tissues. Ferritin shared strong variants with an *APOE* plasma pQTL at 19q13, while delirium mapped to a distinct cortical regulatory signal at *CEACAM19* within the same locus. Beyond 19q13, ferritin colocalized with *SLC11A2*/*DMT1* in whole-blood eQTL and *TF* in liver sQTL, both known mediators in the iron pathway [6, 22–24], without sharing variants with delirium. This pattern supports a model in which the inherited component captured by systemic ferritin-related variation is genetically distinct from the component captured by delirium susceptibility signals in these datasets, rather than indicating a shared genome-wide genetic basis.

Recent perioperative studies continue to report links between preoperative ferritin and postoperative delirium in older adults, reinforcing the clinical importance of the association but leaving causality unresolved [25]. An important consideration is that ferritin’s interpretability depends on timescale and context; ferritin reflects iron stores and also serves as an acute-phase reactant during infection, trauma, and inflammation, driven by cytokine-hepcidin programs that restrict iron availability and alter iron trafficking [6–8, 10]. Similarly, our results suggest elevated ferritin during illness may result from precipitating factors (e.g., infection, trauma, surgery, medication) rather than from baseline vulnerability [1, 2]. While biomarker studies of ICU delirium often identify peripheral markers reflecting systemic injury or inflammation, these findings are difficult to translate into mechanisms or actionable prevention strategies [26]. Therefore, our findings argue against baseline genetically influenced systemic ferritin as a major shared genetic driver or preventive target for delirium, although ferritin may still remain clinically informative as a marker of acute stress during illness.

At 19q13, ferritin showed a strong variant sharing with the *APOE* plasma pQTL (PPH4 = 0.999), while delirium shared a different signal, linked to a distinct cortical regulatory region at *CEACAM19*, despite physical proximity. This dissociation is consistent with the recognized multiple-signal complexity of the *APOE/TOMM40* region and illustrates that adjacent molecular QTLs can represent distinct causal variants and pathways rather than a single pleiotropic effect [17, 27]. Practically, the detailed locus architecture supports two main interpretations: (a) systemic coupling of ferritin with *APOE*-linked circulating proteins, and (b) a brain-specific regulatory vulnerability for delirium that is separate from circulating ApoE levels.

Mechanistically, the lack of shared signals between delirium and *APOE* protein or *APOE* brain sQTLs does not rule out neuroimmune roles in the *APOE* region, despite suggesting that the inherited genetic component captured by these molecular markers is not the primary factor driving delirium susceptibility in these datasets. *APOE* participates in microglial state-transition programs and neuroinflammatory responses (including the *TREM2*-*APOE* axis), which influence responses to injury and metabolic stress, offering a plausible neurobiological rationale for delirium vulnerability, even though the shared variants differ [27, 28]. Of particular interest is a cortical eQTL signal implicating *CEACAM19*, since *CEACAM* proteins are involved in cell adhesion and signaling and play essential roles in immune processes [29]. Although not a typical delirium gene, its regulatory link in the cortex aligns with current delirium conceptualizations in which vulnerability depends on glial-vascular interactions and on cortical networks responding to systemic inflammation and metabolic stress; thus, regulatory variation affecting adhesion signaling could modulate network stability without implicating systemic iron mechanisms.

On the exposure side, colocalization of ferritin with *SLC11A2*/*DMT1* in blood and transferrin (*TF*) in the liver supports the biological interpretability of the ferritin genetic instruments. *SLC11A2*/*DMT1* is a principal importer of ferrous iron at plasma and endosomal membranes, while *TF* is essential for iron transport and hepatic feedback regulation via hepcidin [6, 23, 24]. This alignment indicates that the null results linking ferritin and delirium are unlikely to be due to uninformative instruments; instead, the instruments appear to reflect expected iron-related biology. The lack of shared loci between ferritin and delirium suggests that systemic iron regulation likely has a limited influence on delirium risk, possibly because brain iron homeostasis and inflammatory responses involve bottlenecks such as blood–brain barrier transport, microglial iron buffering, and compartmentalized iron handling, insulating baseline systemic variations from the acute neuroimmune cascades that precipitate delirium [2, 4, 22].

### Limitations

Several limitations influence the interpretation of these findings. First, because delirium has a heterogeneous definition, delirium ascertainment via the ICD-10 F05 code has high specificity but low sensitivity in hospital data, potentially biasing effect estimates toward the null and reducing power for gene-mediator detection in SMR. Second, by design, genetic instruments proxy for lifelong ferritin levels, not the acute-phase elevation seen during systemic illness; therefore, context-specific causal effects of acute iron dysregulation cannot be excluded by these analyses. Third, current brain QTL datasets are underpowered, and inconsistent variant overlap across platforms limits the accuracy of fine-mapping at some loci. Lastly, we limited analyses to predominantly European-ancestry datasets and LD references, which enhances internal consistency but reduces generalizability. These constraints emphasize the need for larger delirium case series, improved phenotyping, and higher-resolution trans-ancestry fine mapping.

## Conclusions

This study’s findings support a model in which serum ferritin is clinically informative as a stress/inflammation biomarker in delirium-precipitating illness, yet inherited baseline ferritin set-points show limited evidence of being an actionable cause of delirium vulnerability. Instead, our results favor a locus- and tissue-specific model, in which systemic ferritin variation is shaped by known systemic iron mediators (e.g., *SLC11A2*,* TF*) and shares a systemic *APOE* protein signal at 19q13, while delirium maps to a separate cortical regulatory signal at *CEACAM19* in the same region. This indicates that, within the genetic architecture captured here, delirium susceptibility is more consistent with brain-specific regulatory signals within a multi-signal locus than with systemic iron handling signals. Thus, our findings highlight the importance of focusing on brain-relevant regulatory pathways that may determine susceptibility to acute neuro-cognitive disruptions under systemic stress. Future research should aim for architecture-aware replication, incorporate cell-type-specific regulatory data, and test mechanistic hypotheses using brain-relevant models that can simulate neuroimmune signalling and network instability.

## Supplementary Information

Below is the link to the electronic supplementary material.

Supplementary Material 1. Supplementary Figures S1–S12. A compiled PDF containing Supplementary Figures S1–S12 supporting the main analyses, including diagnostic and sensitivity plots, SMR/HEIDI summaries across panels, and locus-level colocalization visualizations.

Supplementary Material 2. Supplementary Tables S1–S20. An Excel workbook containing Supplementary Tables S1–S20 (one worksheet per table) reporting detailed numeric results underlying the manuscript, including MR summary outputs, SMR/HEIDI results across QTL panels, and colocalization/SuSiE summary statistics.

Supplementary Material 3. Supplementary legends (Tables and Figures). A Word document providing the full captions/legends for all supplementary tables (S1–S20) and supplementary figures (S1–S12), including brief methodological notes needed to interpret the supplementary items.

## Data Availability

Ferritin GWAS summary statistics are available via the GWAS Catalog (accession GCST90270865), and delirium GWAS summary statistics are available via the GWAS Catalog (accession GCST90473243). The QTL summary datasets used for SMR/HEIDI and colocalization analyses (eQTL/sQTL/pQTL/mQTL panels, including GTEx, eQTLGen, OpenGWAS pQTL resources, and GoDMC mQTL resources) are publicly available from their respective providers and were accessed under the terms specified by those resources. All analysis code, processed outputs, and an interactive supplementary HTML are available in the Zenodo repository:(10.5281/zenodo.18135973). The development repository is available at (https://github.com/amjahromizadeh/Ferritin-Delirium).

## Abbreviations

BBB: Blood–brain barrier
CEACAM19: Carcinoembryonic antigen-related cell adhesion molecule 19
CI: Confidence interval
eQTL: Expression quantitative trait locus
GWAS: Genome-wide association study
HEIDI: Heterogeneity in dependent instruments
IRNT: Inverse-rank-normalized transformation
IVW: Inverse-variance weighted
LD: Linkage disequilibrium
LDSC: Linkage disequilibrium score regression
MAF: Minor allele frequency
MR: Mendelian randomization
MR-PRESSO: Mendelian randomization Pleiotropy RESidual Sum and Outlier
MR-RAPS: Mendelian randomization robust adjusted profile score
mQTL: Methylation quantitative trait locus
OR: Odds ratio
pQTL: Protein quantitative trait locus
QC: Quality control
QTL: Quantitative trait locus
SE: Standard error
SMR: Summary-data-based Mendelian randomization
SNP: Single-nucleotide polymorphism
sQTL: Splicing quantitative trait locus
SuSiE: Sum of single effects
WGS: Whole-genome sequencing
