# Supplementary material for: Serum ferritin and delirium risk: an integrative genomic analysis of causal inference and multi-tissue regulatory signals
Source: Hum Genomics. 2026 Apr 25;20:97. doi: 10.1186/s40246-026-00972-5 (PMC13262423; doi:10.1186/s40246-026-00972-5)

Figure S1. Causal estimates across MR estimators. OR= Odds Ratio.Estimates are directionally consistent; primary IVW (randomeffects) interval includes the null.

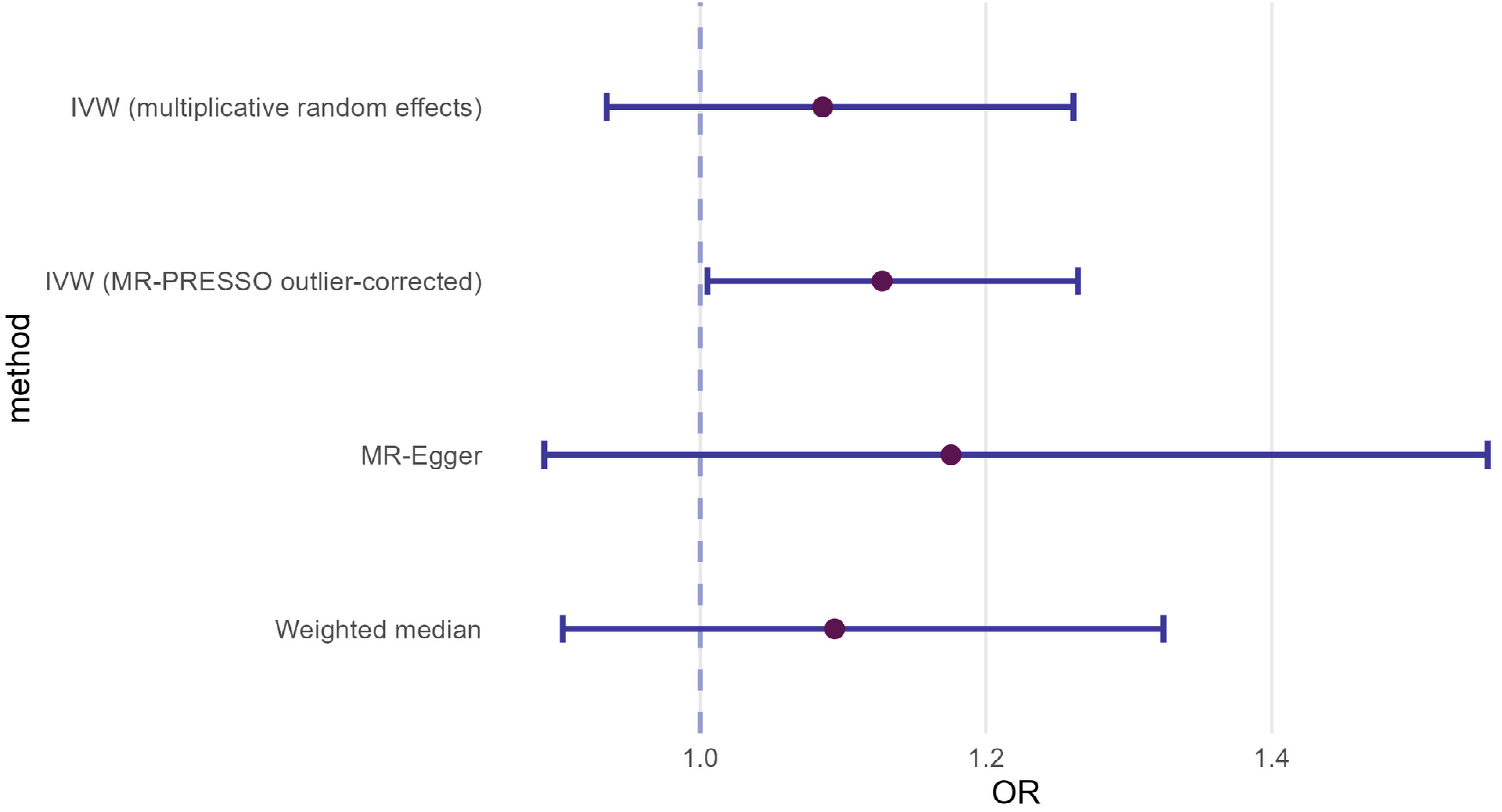

Figure S2. MR scatter plot. Scatter of per-SNP effects (ferritin vs delirium) with estimator lines; approximate alignment of slopes supports method-level consistency in direction.

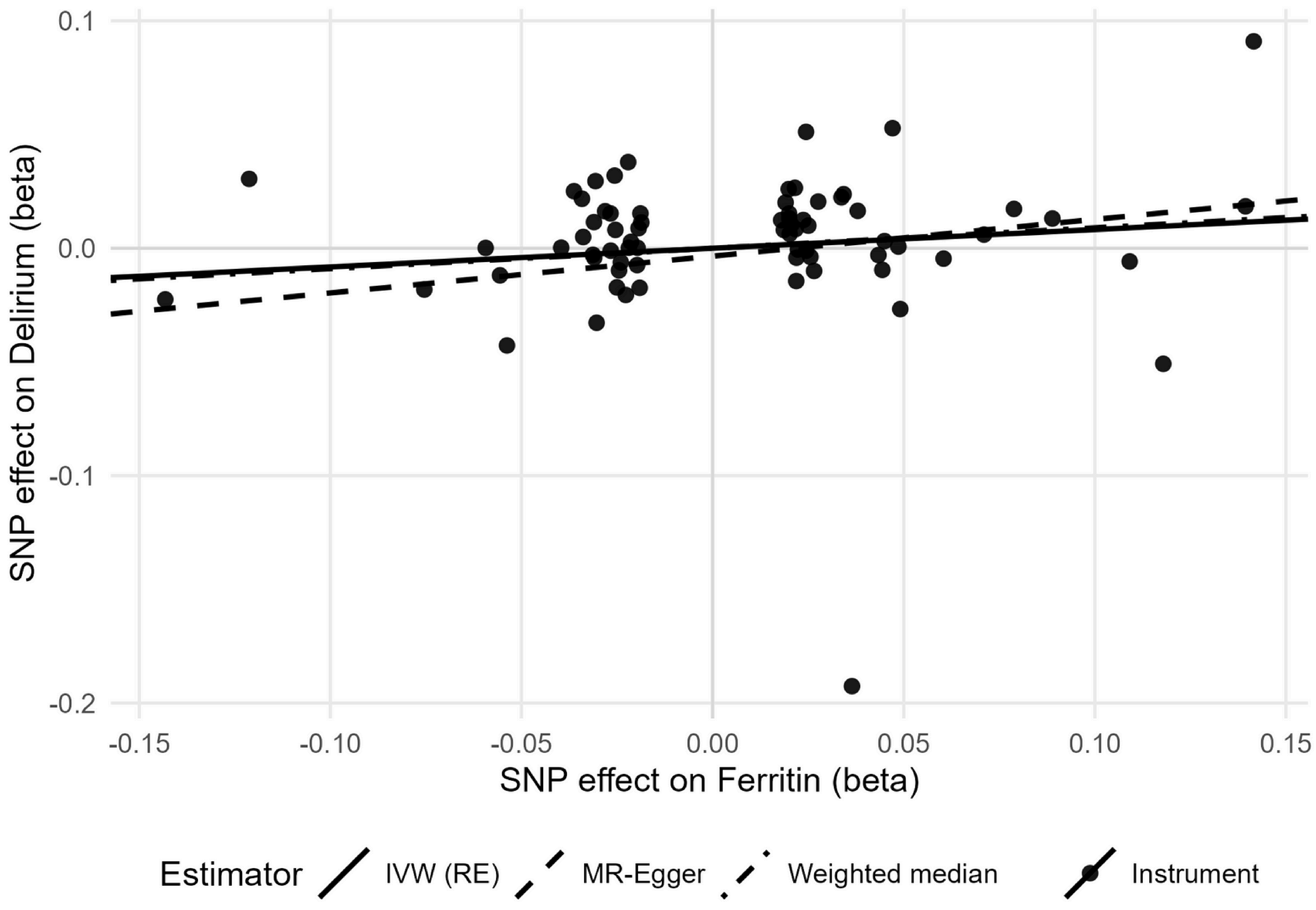

Figure S3. Funnel plot. Funnel plot of per-SNP Wald ratio estimates versus their standard errors to screen for outliers.

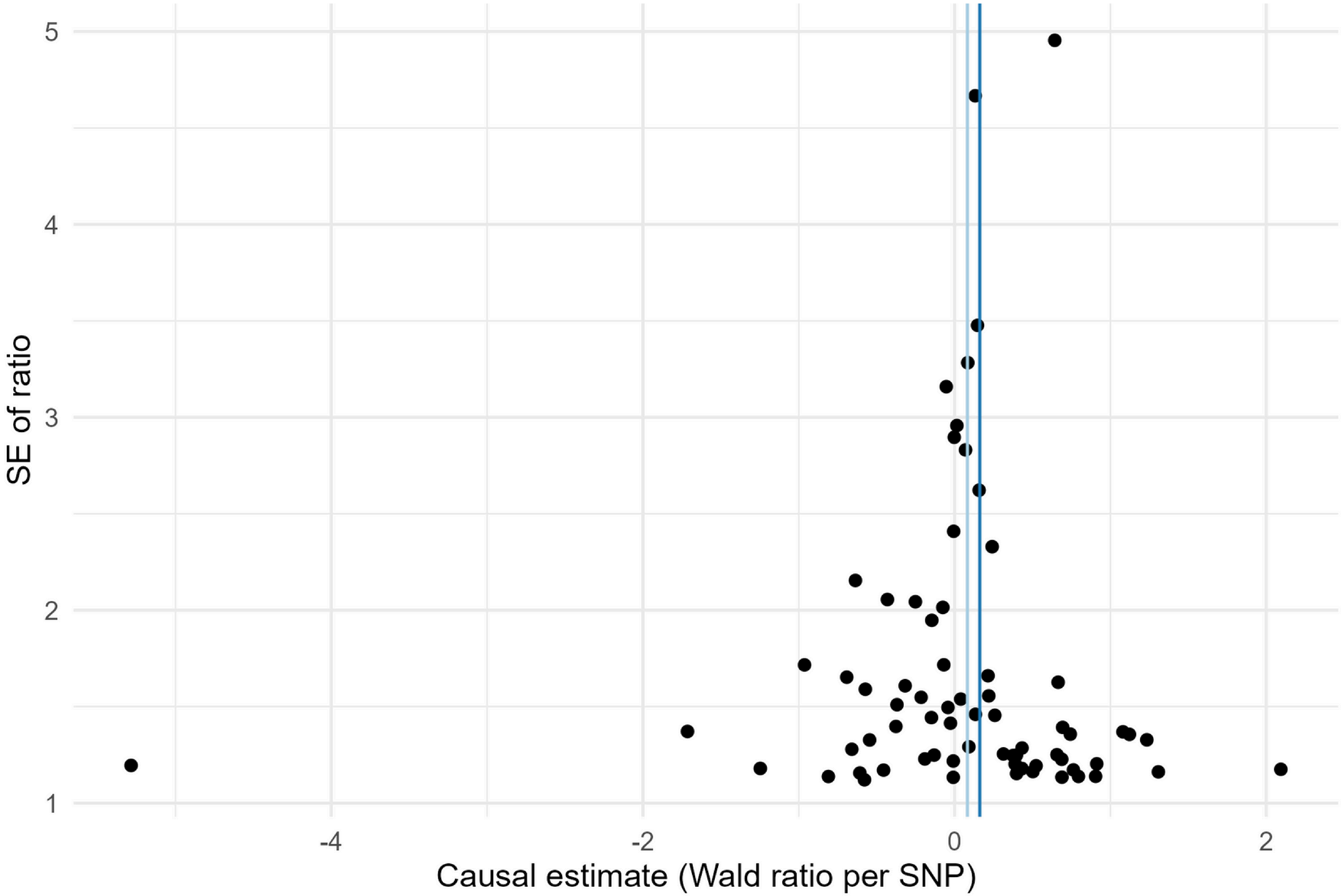

Figure S4. Leave-one-out influence plot. Stability of the IVW estimate under leave-one-out suggests the inference is not driven by a single SNP.

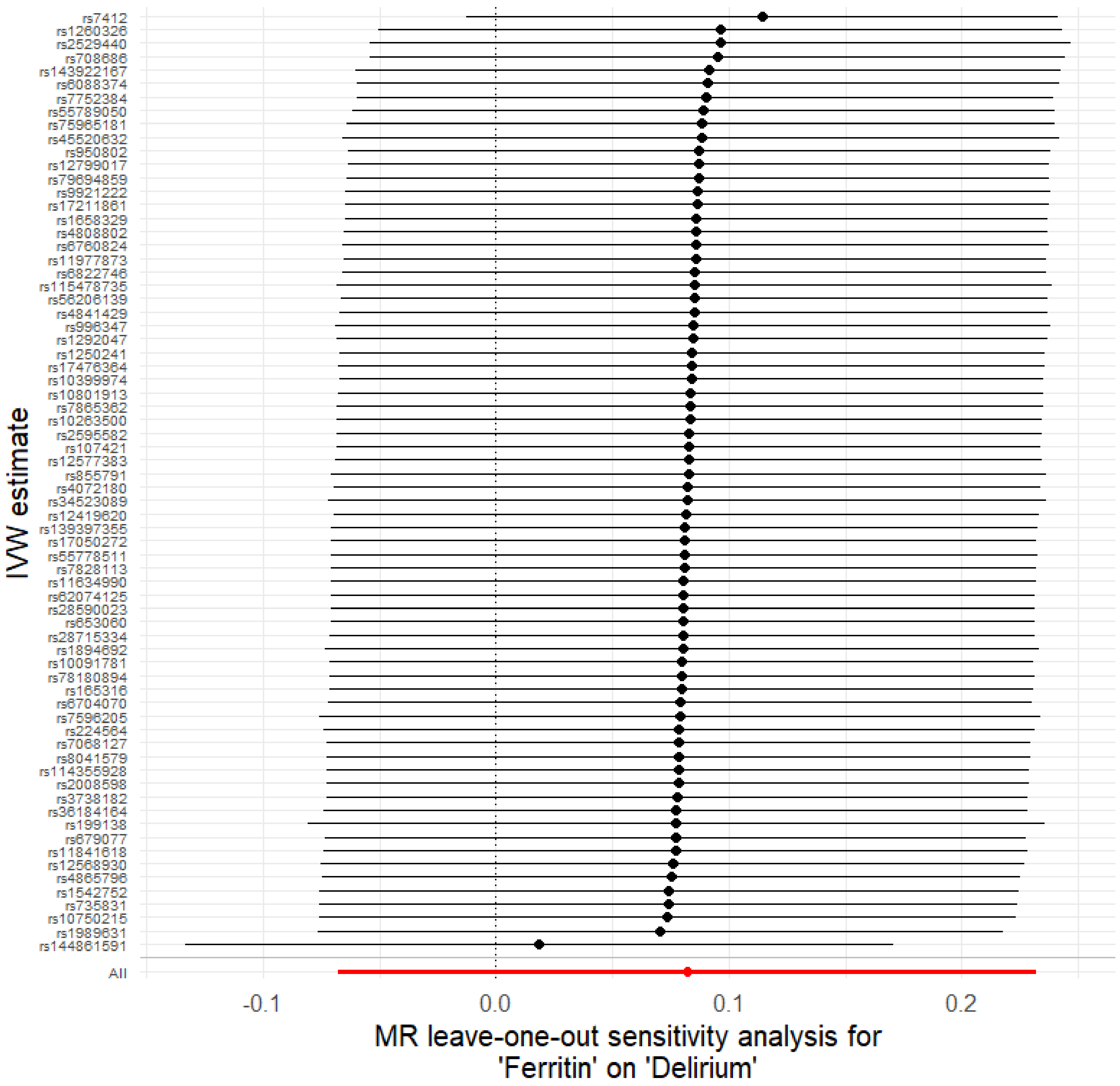

Figure S5. MR-PRESSO outlier plot. MR-PRESSO outlier map showing studentized residuals versus leverage (hat values) for instruments, with global and distortion test p-values and identified outliers.

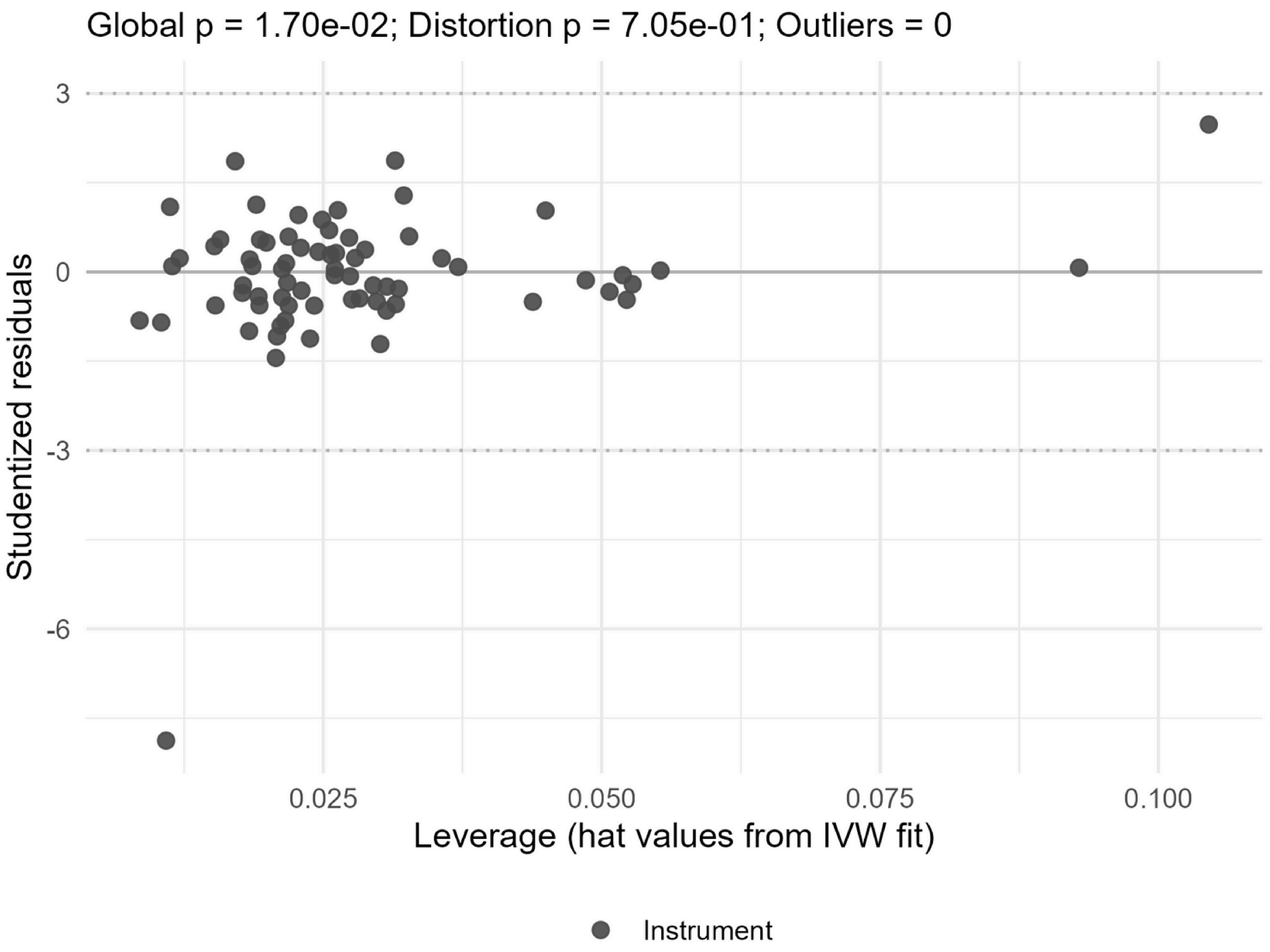

Figure S6. Tissue × omics yield heatmap (sum of BF+HEIDI passing probes across panels). Heatmap of aggregated BF+HEIDI passing probe counts across panels by tissue and omics layer, shown separately for Delirium, on the left, and Ferritin, on the right.

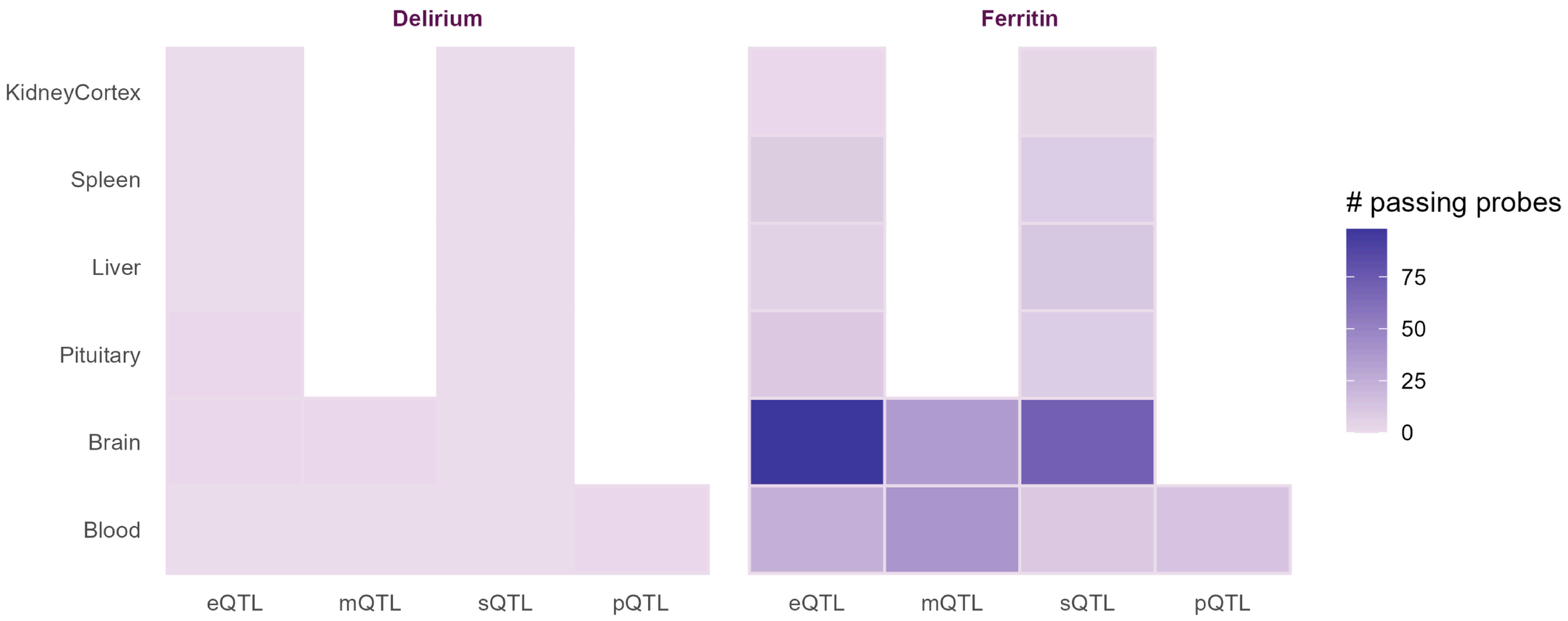

Figure S7. Highest-yield Ferritin panels by number of BF+HEIDI passing probes. Ferritin cis-QTL panels ranked by the number of BF+HEIDI passing probes. Horizontal bars represent passing probe counts per xQTL panel.

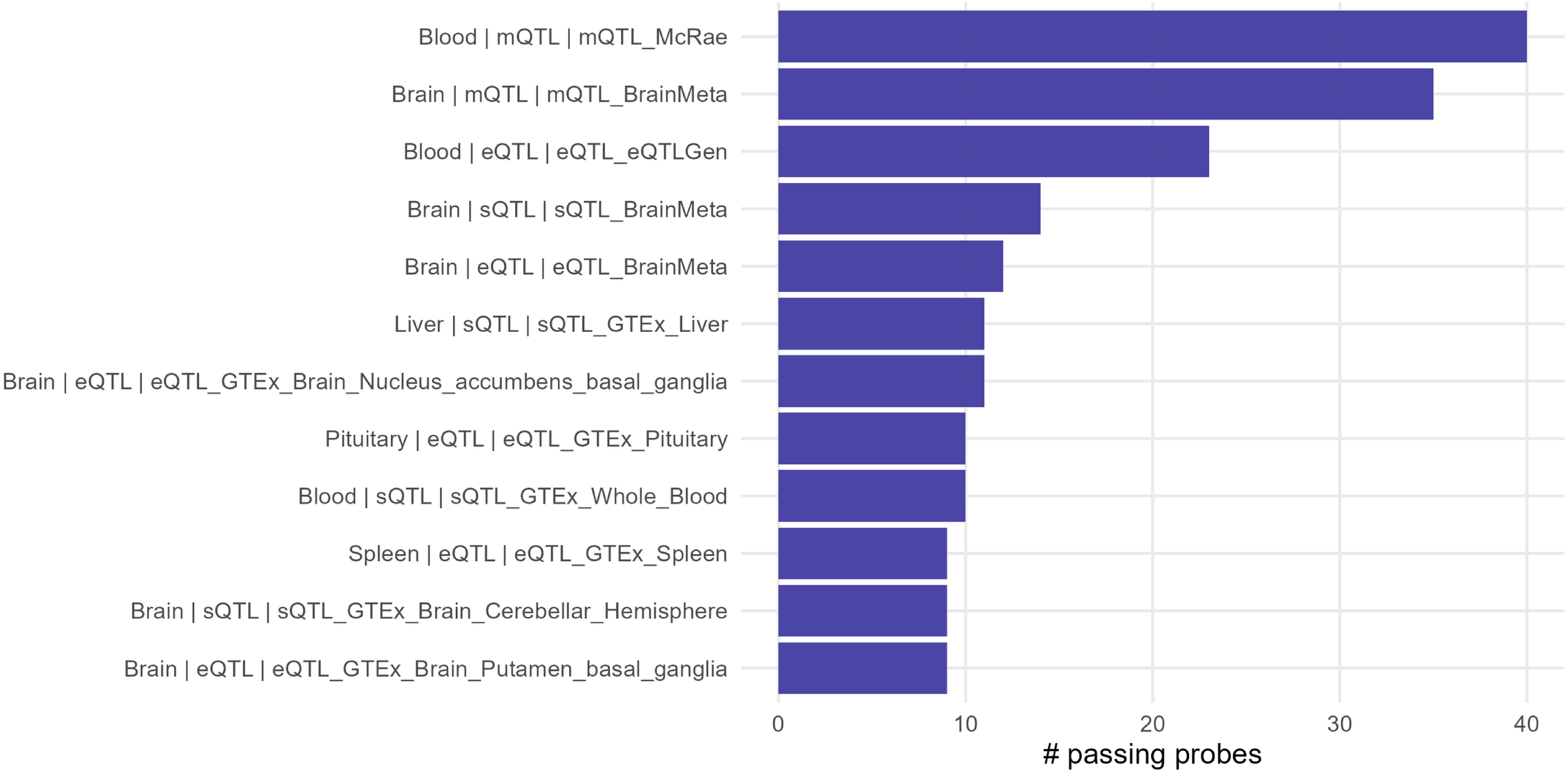

Figure S8. Top Ferritin genes based on SMR significance. Top Ferritin genes ranked by the most significant SMR association per gene (minimum p_{SMR} across BF+HEIDI passing records).

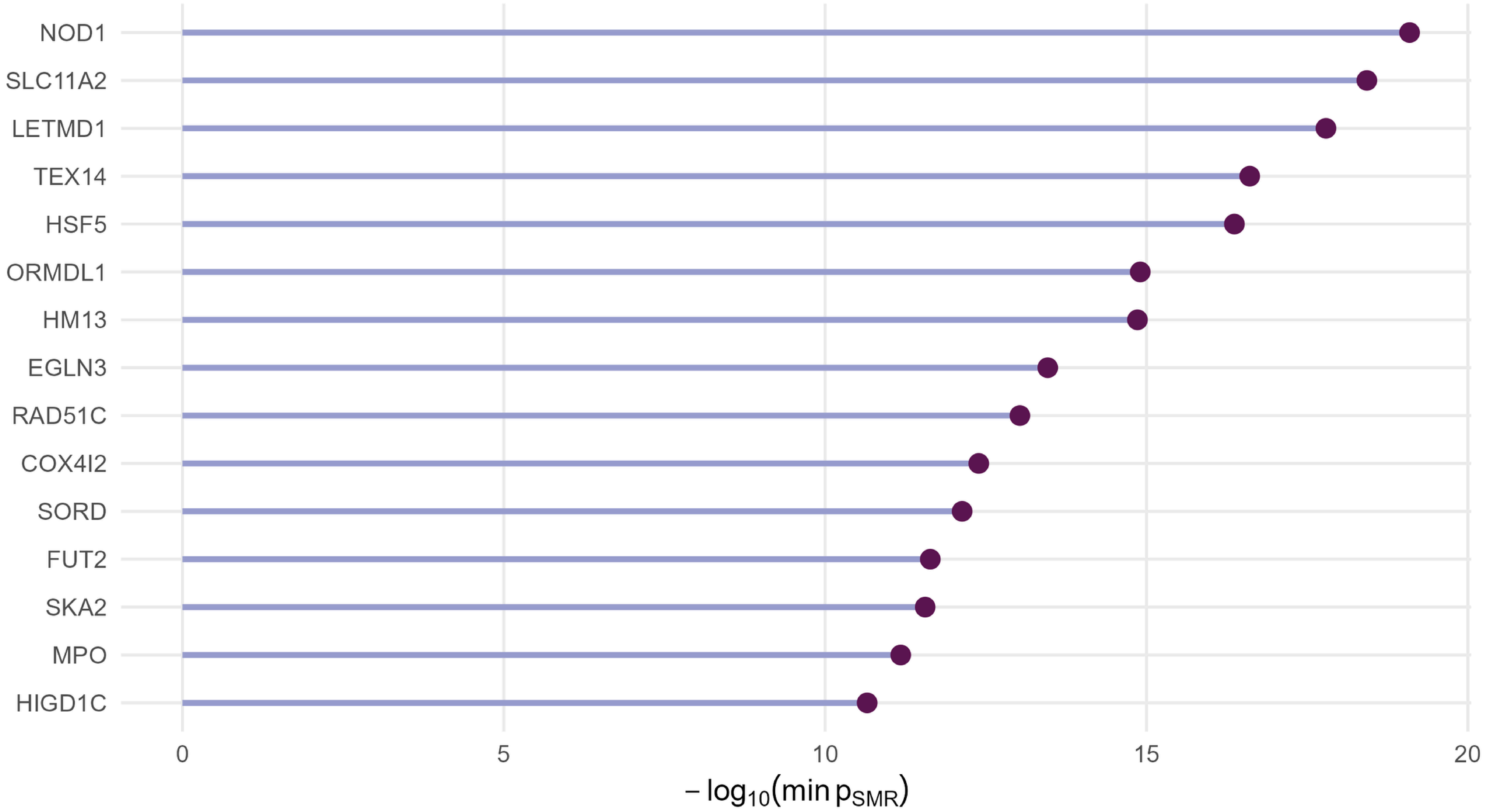

Figure S9. Delirium retained genes (best pSMR per gene). All three signals are located at the 19q13 locus.

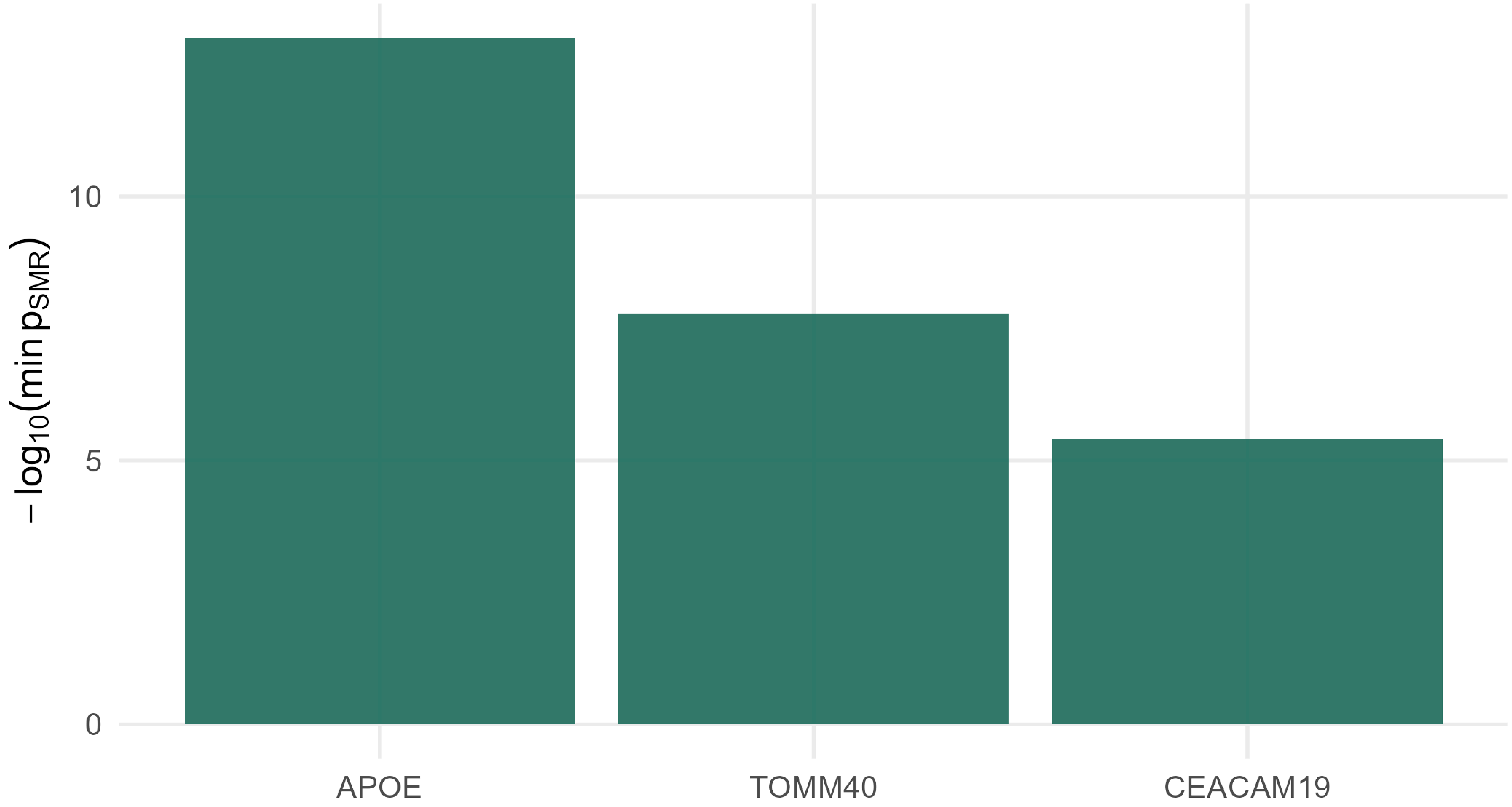

Figure S10. Colocalisation evidence across key tests (PP.H4). Posterior probability of a shared causal variant (PP.H4) across key GWAS–QTL/proxy comparisons. The dashed line marks the PP.H4 ≥ 0.80 threshold to denote strong shared-signal support.

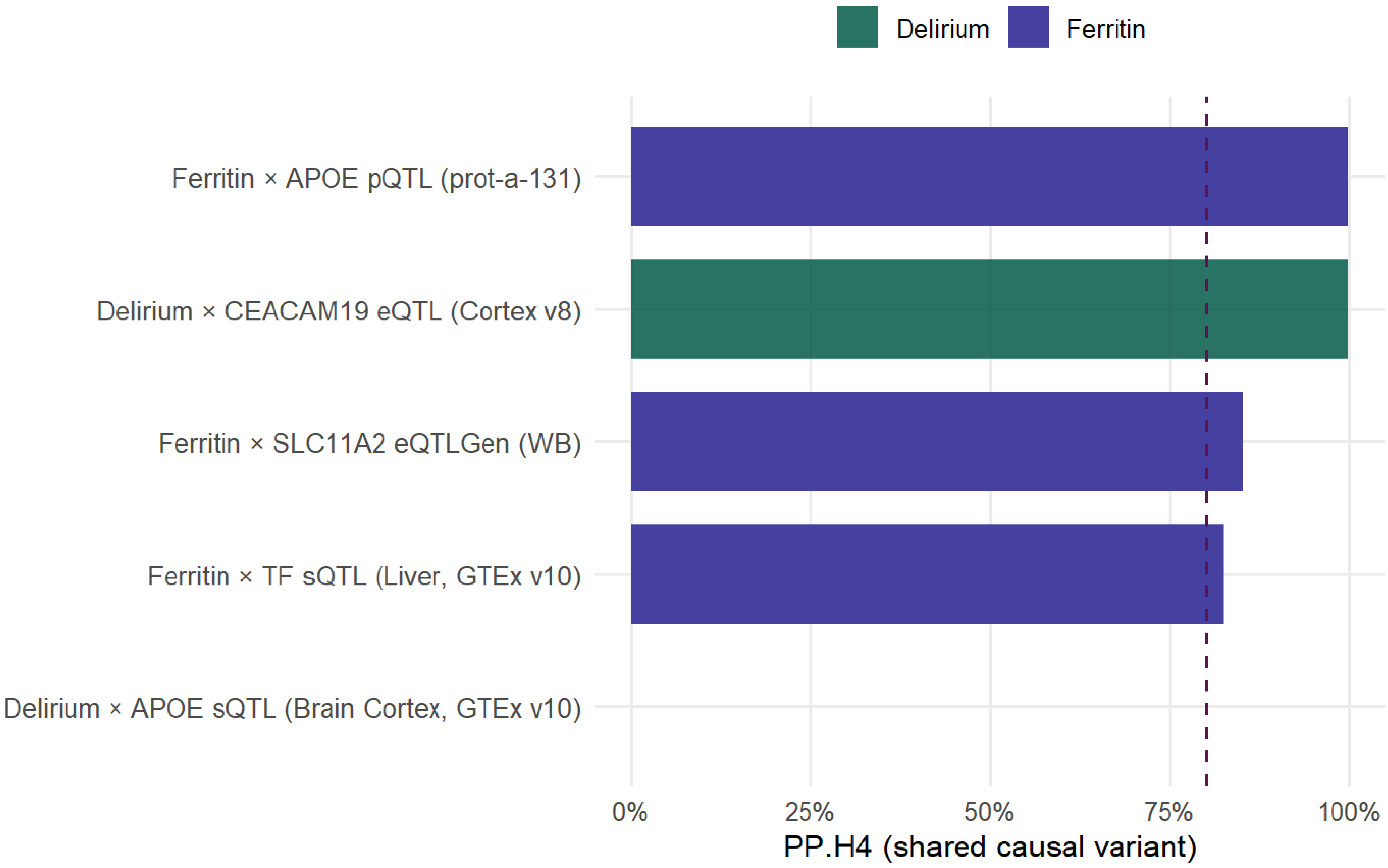

Figure S11. Component-pair heatmap. SuSiE–coloc component-pair matrix showing PP.H4 support for shared signals between Ferritin GWAS components and APOE pQTL components. Darker cells indicate stronger colocalisation.

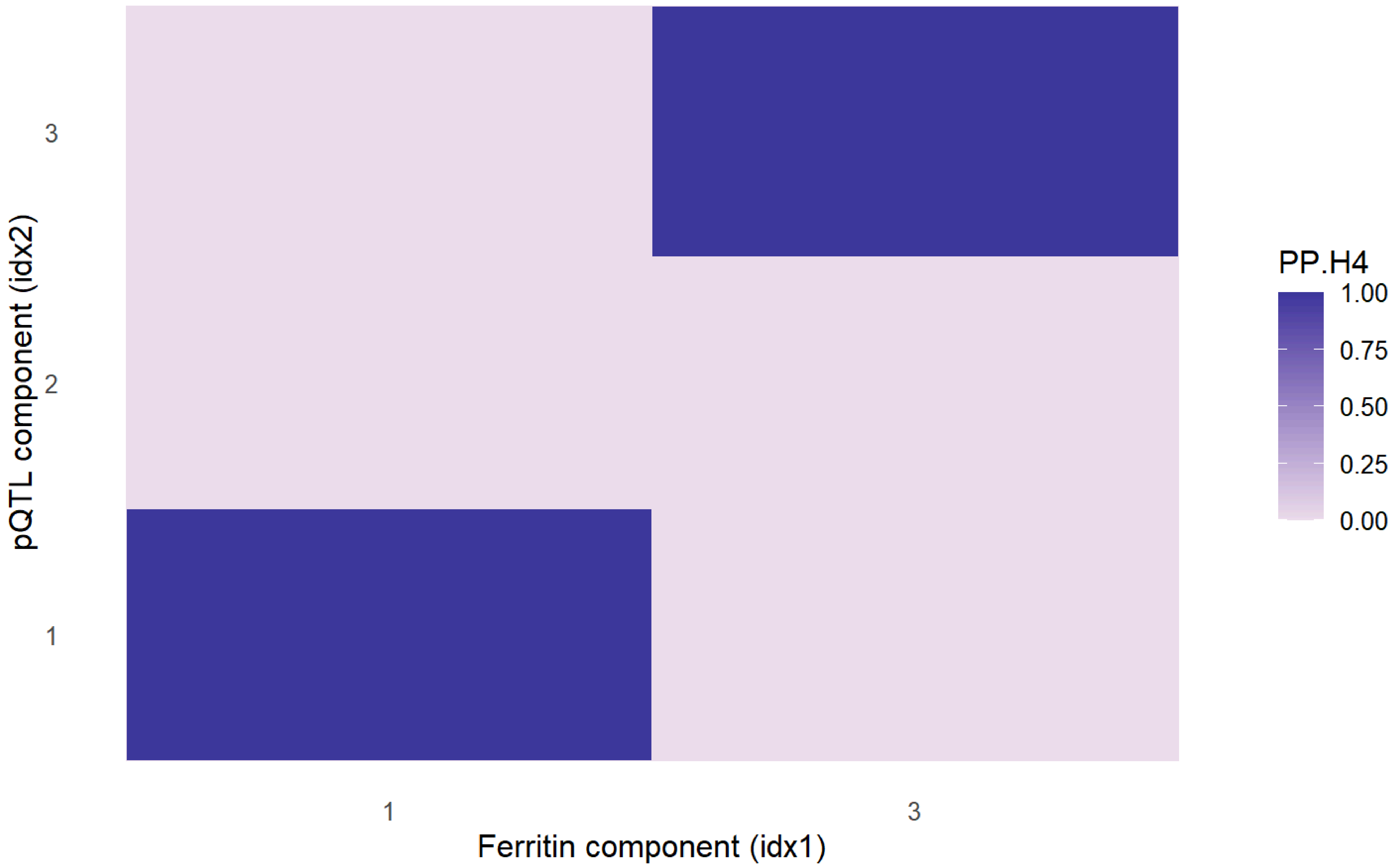

Figure S12. Per-SNP PP.H4 at the CEACAM19 locus. SNP-level PP.H4 contributions for Delirium × CEACAM19 cortex colocalisation, highlighting the variants that drive the shared-signal posterior in this region.

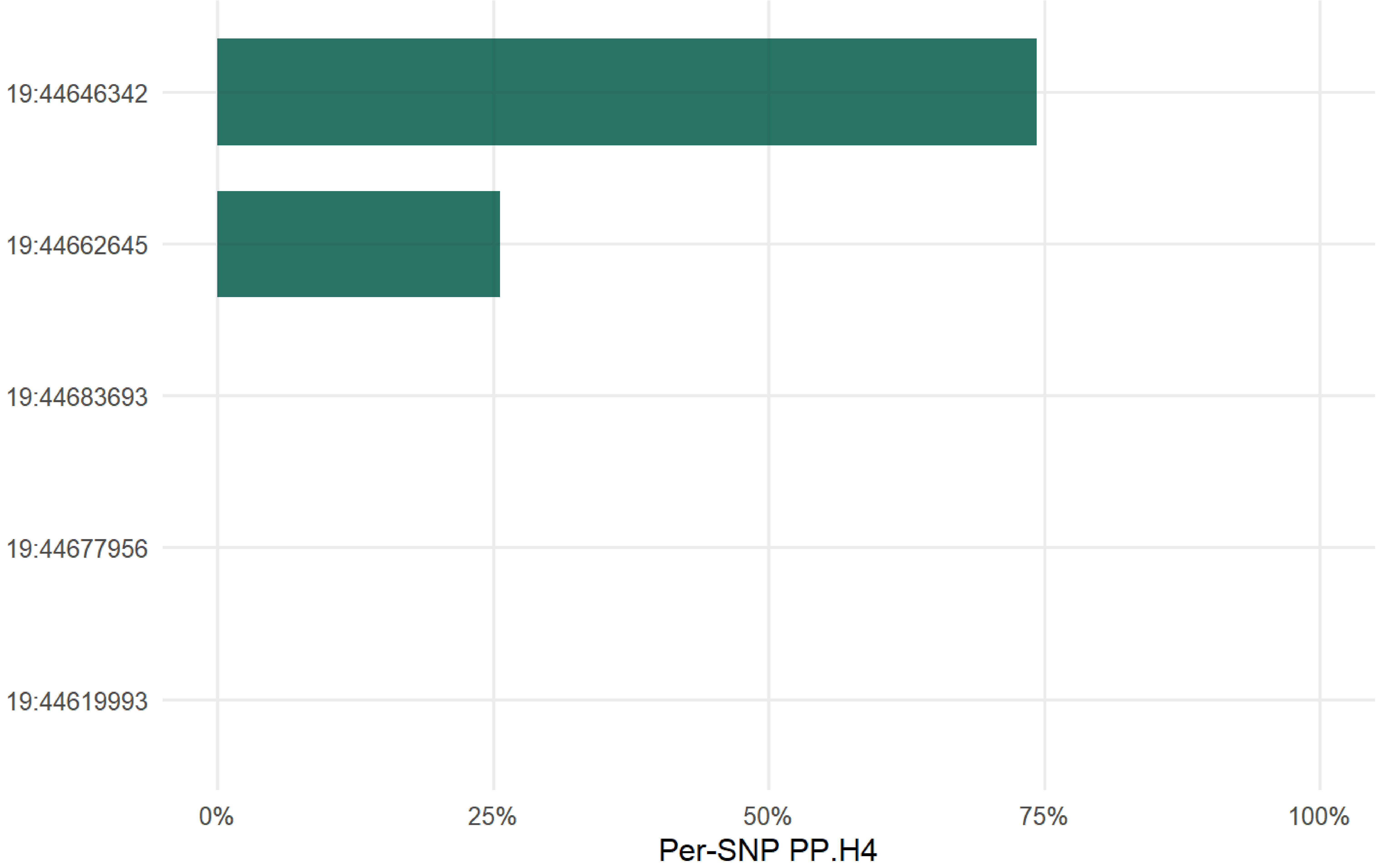

Supplement: Supplementary file 1 — Supplementary Material 1. Supplementary Figures S1–S12. A compiled PDF containing Supplementary Figures S1–S12 supporting the main analyses, including diagnostic and sensitivity plots, SMR/HEIDI summaries across panels, and locus-level colocalization visualizations. [file 40246_2026_972_MOESM1_ESM.pdf]
