# Supplementary material for: Serum ferritin and delirium risk: an integrative genomic analysis of causal inference and multi-tissue regulatory signals
Source: Hum Genomics. 2026 Apr 25;20:97. doi: 10.1186/s40246-026-00972-5 (PMC13262423; doi:10.1186/s40246-026-00972-5)
Supplement: Supplementary file 3 — Supplementary Material 3. Supplementary legends (Tables and Figures). A Word document providing the full captions/legends for all supplementary tables (S1–S20) and supplementary figures (S1–S12), including brief methodological notes needed to interpret the supplementary items. [file 40246_2026_972_MOESM3_ESM.docx]

**Table S1.** Two-sample MR causal estimates for ferritin → delirium across IVW, MR-Egger, and weighted median (OR per SD higher ferritin).

**Table S2.** Instrument diagnostics for ferritin → delirium, including strength (F), heterogeneity (Cochran’s Q), directional pleiotropy (Egger intercept), directionality (Steiger), and MR-PRESSO outlier assessment.

**Table S3.** Cochran’s Q heterogeneity statistics for IVW and MR-Egger models (Q, df, p).

**Table S4.** MR-Egger intercept test for directional pleiotropy (intercept, SE, p).

**Table S5.** Steiger filtering results comparing variance explained in exposure vs outcome (R²x, R²y) and inferred causal direction.

**Table S6.** MR-PRESSO raw and outlier-corrected causal estimates for ferritin → delirium, including OR.

**Table S7.** SMR/HEIDI panel-level summary by trait, tissue, omics layer, and QTL panel, reporting probes/genes passing Bonferroni+HEIDI and the most significant retained signal per panel.

**Table S8.** Probe-level SMR hits retained after panel-wise Bonferroni and HEIDI filtering, with lead SNP, effect estimates, and HEIDI diagnostics across traits and panels.

**Table S9.** All Delirium SMR associations retained under Bonferroni+HEIDI, listing locus details, lead SNP, SMR effect, and HEIDI statistics.

**Table S10.** Strict cross-trait overlap of BF+HEIDI-retained genes, showing matching retained records for the shared gene(s) across Ferritin and Delirium.

**Table S11.** Aggregated counts of Bonferroni+HEIDI passing probes by tissue and omics layer, shown separately for Ferritin and Delirium.

**Table S12.** Panel-level SMR/HEIDI results by trait, tissue, omics layer, and QTL panel, reporting the number of tested probes, panel-wise Bonferroni threshold, BF+HEIDI passing counts, and the top retained signal per panel.

**Table S13.** Complete Bonferroni+HEIDI-retained SMR hit list across both traits, including panel metadata, lead SNP, SMR effect/SE, p-values, and HEIDI support.

**Table S14.** Top Ferritin BF+HEIDI passing records ranked by p_SMR_, reporting locus details, lead SNP, SMR effect, and HEIDI statistics.

**Table S15.** Colocalisation posterior summaries across key loci and proxy panels, contrasting evidence for distinct signals (PP.H3) versus a shared causal variant (PP.H4)

**Table S16.** Top SuSiE component-pair matches for Ferritin GWAS × APOE pQTL, ranked by PP.H4.abf, reporting best-aligned SNP pairs and posterior breakdown.

**Table S17.** SuSiE summary for Delirium GWAS × APOE pQTL, listing credible sets, lead SNPs, and the maximum observed shared-signal probability (PP_shared).

**Table S18.** Coloc.abf posterior probabilities (H0–H4) for Delirium GWAS × CEACAM19 eQTL in cortex (v8), as recorded in the runlist.

**Table S19.** SNP-level ranking by PP.H4 for the Delirium × CEACAM19 cortex colocalisation window, highlighting variants driving the shared-signal posterior.

**Table S20.** Lead-variant effect alignment for Delirium GWAS and CEACAM19 cortex eQTL (betas/SE/MAF) alongside PP.H4, supporting interpretation of the colocalised signal.
